# Supplementary material for: Copolymer dielectrics with balanced chain-packing density and surface polarity for high-performance flexible organic electronics
Source: Nat Commun. 2018 Jun 14;9:2339. doi: 10.1038/s41467-018-04665-z (PMC6002412; doi:10.1038/s41467-018-04665-z)
Supplement: Supplementary file 1 — Supplementary Information [file 41467_2018_4665_MOESM1_ESM.pdf]

Supplementary Information

Copolymer Dielectrics with Balanced Chain-Packing Density and Surface Polarity for High-Performance Flexible Organic Electronics

Ji et al.

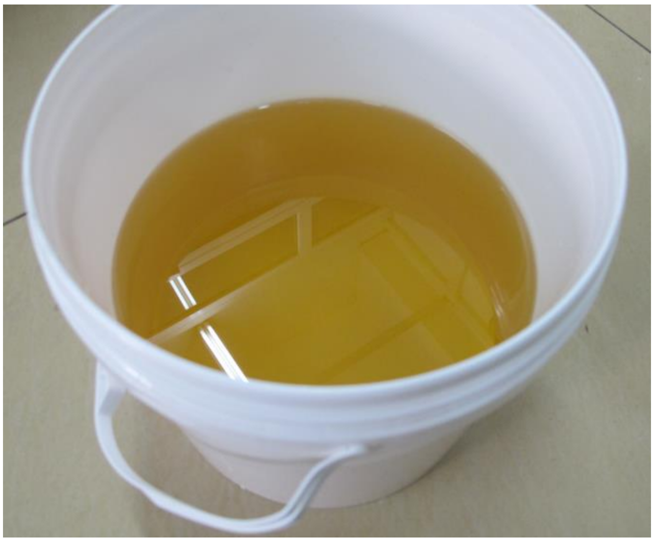

Supplementary Figure 1 | The solution of precursor poly (amic acid) (PAA). The precursor poly (amic acid) (PAA) can be mass produced (3 L) by polymerizing pyromellitic dianhydride (PMDA, $C_{10}H_2O_6$) and 4, 4'-Oxydianiline (ODA, $C_{12}H_{12}N_2O$).

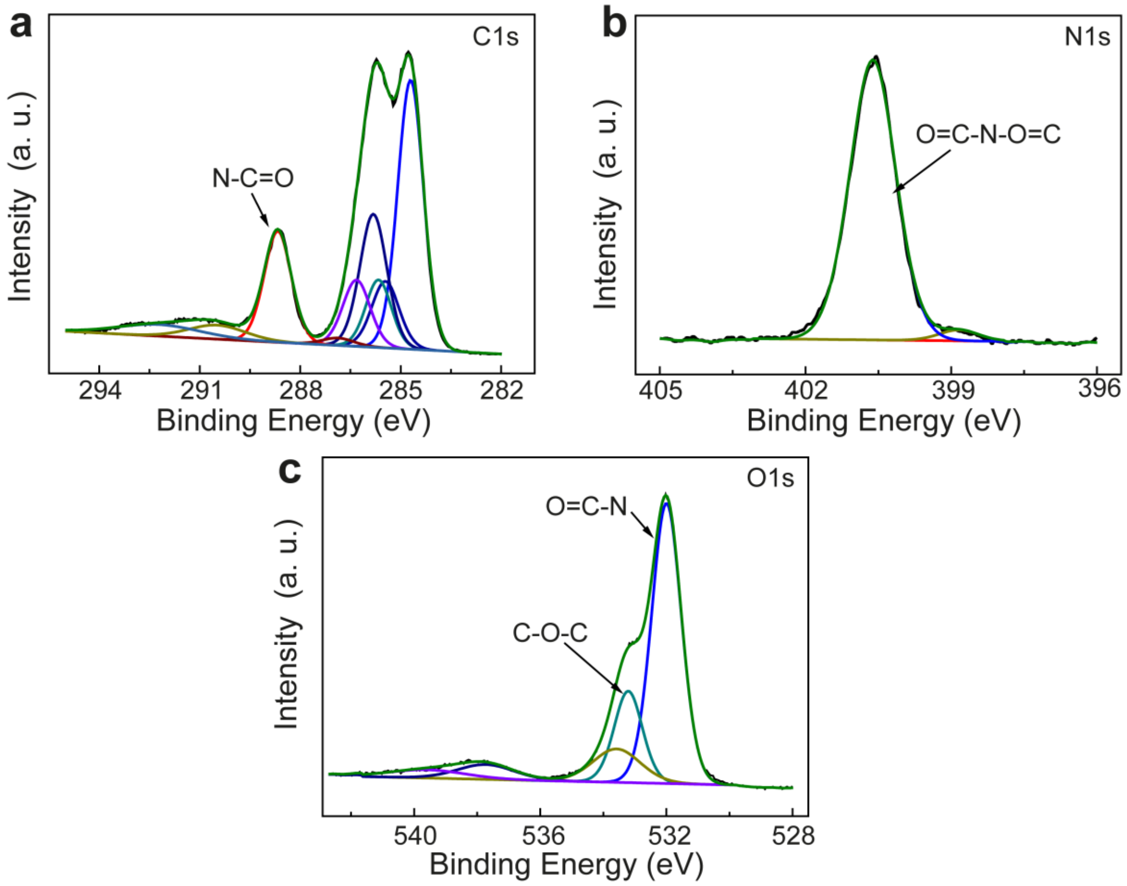

Supplementary Figure 2 | X-ray photoelectron spectroscopy (XPS) C ls, N ls and O ls spectra of polyimide. X-ray photoelectron spectroscopy (XPS) C ls (**a**), N ls (**b**) and O ls (**c**) spectra of polyimide. Only imide groups (O=C-N-C=O, with C 1s, N 1s and O 1s peaks located at ~288.4, ~400.6 and ~531.7 eV, respectively) were detected on fully (100%) imidized polyimide surface.

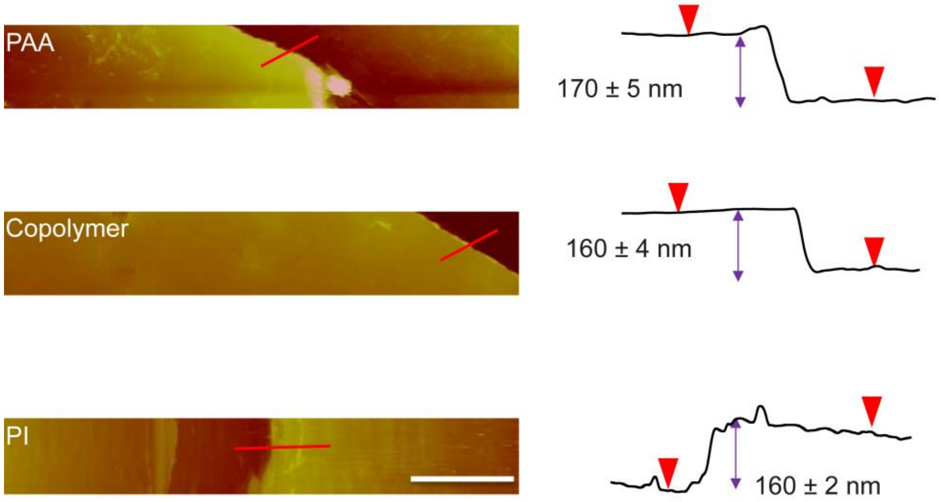

Supplementary Figure 3 | The AFM measurement of the thickness of dielectrics. The AFM images of dielectric films with the thickness around 160 nm. The PAA was spin-coated on the ITO substrate to form different dielectric layers after being treated under different annealing temperatures (PAA, 25 ^{o}C; copolymer, 200 ^{o}C and PI, 300 ^{o}C). Scale bar, 4 μm.

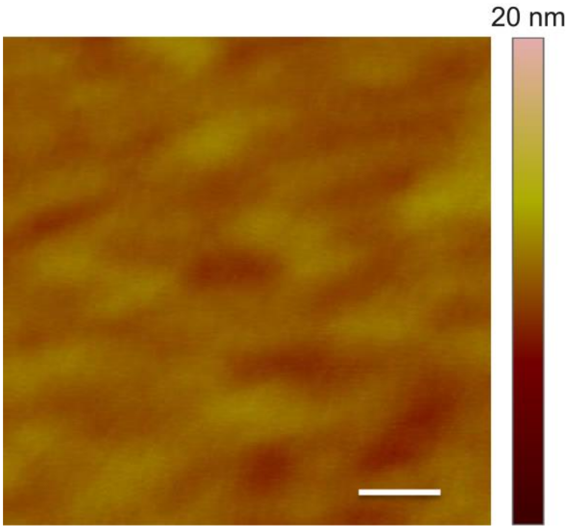

Supplementary Figure 4 | The AFM measurement of the surface of copolymer. The AFM image of copolymer film with the thickness of 160 nm. This copolymer film could be easily fabricated by spin-coating the solution of PAA on the surface of ITO and then annealed at the temperature of 200 ^{o}C. Scale bar, 600 nm.

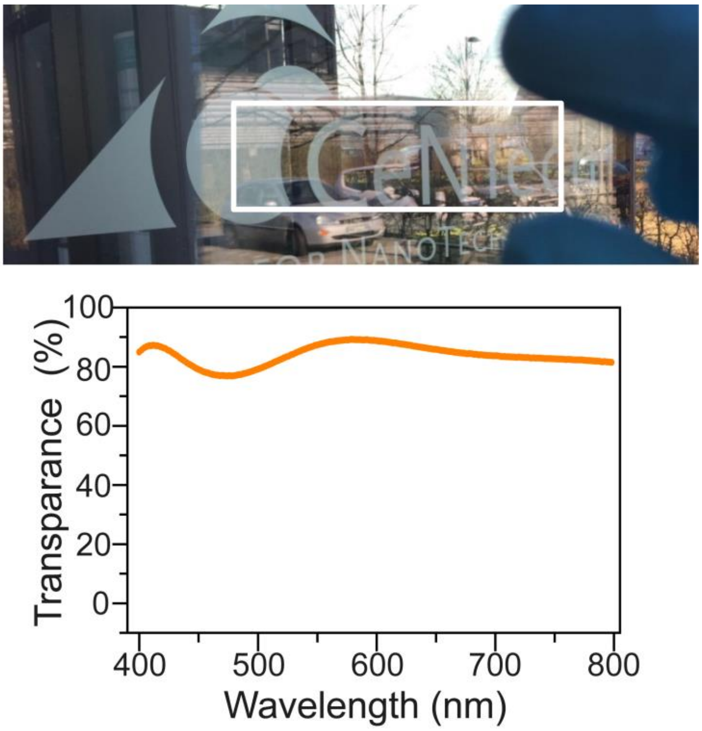

Supplementary Figure 5 | The optical characterization of copolymer film. The logo “CeNTech” under the transparent copolymer film and the transmission spectrum of this copolymer film. Large-area copolymer film could be easily produced and this film was transparent (the logo “CeNTech” could be clearly observed) with an excellent transparency of > 80% in the visible region.

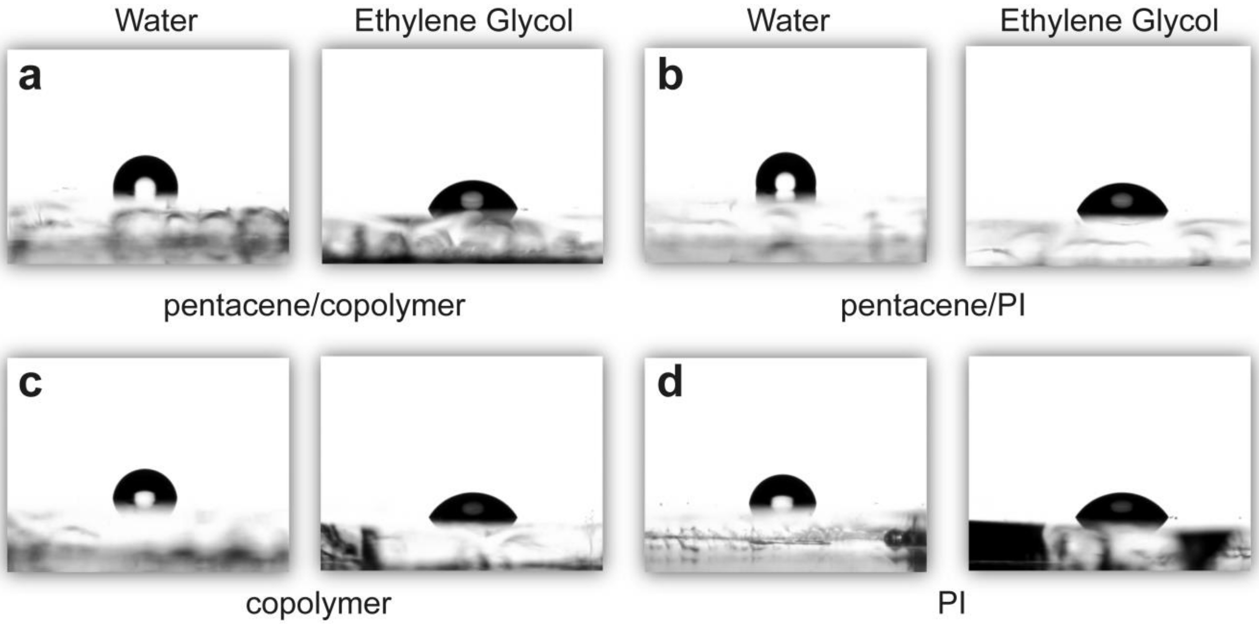

Supplementary Figure 6 | Surface energy measurements. Contact angle on the surface (**a**) pentacene on copolymer; (**b**) pentacene on PI; (**c**) copolymer; (**d**) PI.

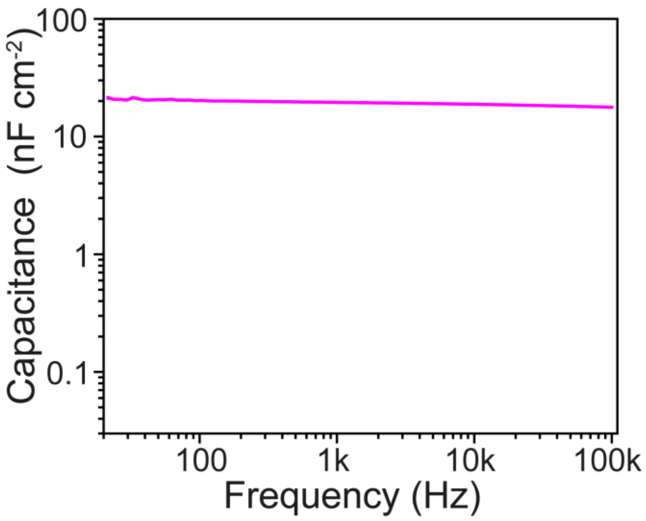

Supplementary Figure 7 | The frequency dependence of capacitance for copolymer dielectrics under 200 ^{o}C annealing temperature. The frequency dependence of capacitance for copolymer dielectrics measured from the sandwich structure (Au/insulator/ITO) showing a capacitance of 20 nF cm^{-2} (at 20 Hz) under 200 ^{o}C annealing.

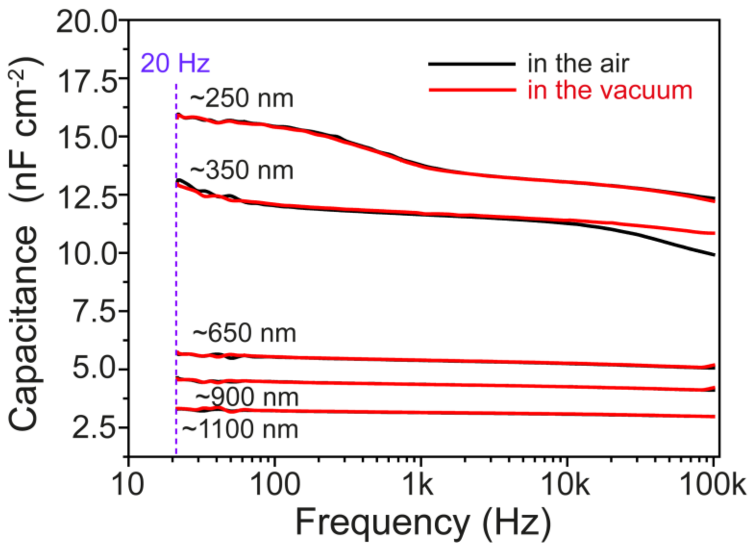

Supplementary Figure 8 | The frequency dependence of capacitance for copolymer dielectrics with different thickness under 200 °C annealing temperature. The capacitance per unit area of copolymer thin film with different thickness under 200 °C annealing temperature is measured (from 20 Hz to 100 kHz) from the sandwich structure (Au/insulator/ITO) in the air (under relative humidity of ~80%-90%) and in the vacuum. Negligible difference of capacitance was observed in the air and in the vacuum, even in the low frequency region.

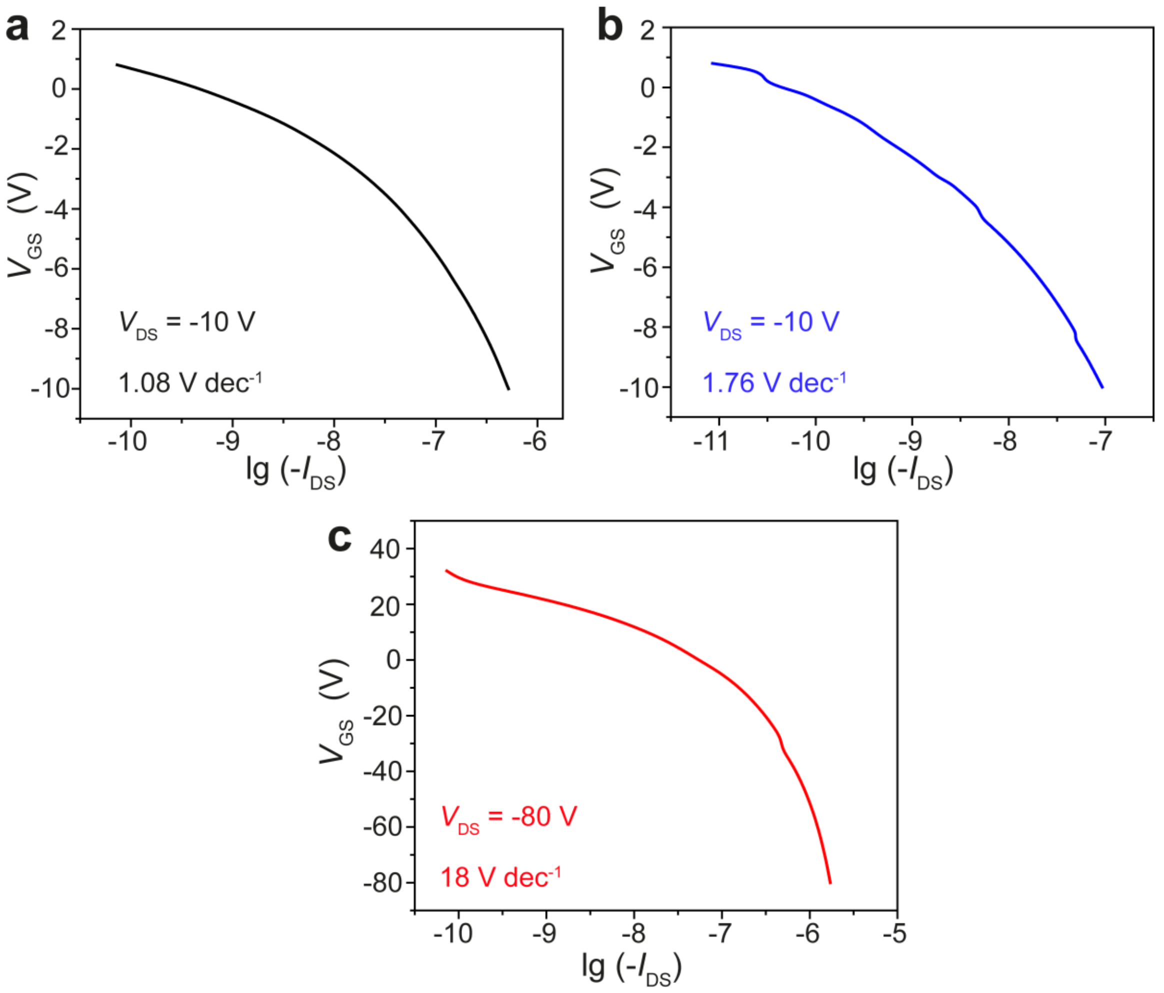

Supplementary Figure 9 | The measurements of the interface trap density. (**a**) 160 nm PI, (**b**) 50 nm SiO_2 and (**c**) 300 nm SiO_2. For comparison, other three dielectric layers (160 nm PI, 50 nm SiO_2 and 300 nm SiO_2) were chosen for OTFT measurements to calculate their interface trap densities. 50 nm-thick pentacene was deposited on these insulators. From the curve of lg ($-I_{DS}$)-V_{GS}, the interface trap densities were 2. 63 $\times 10^{12}$ cm^{-2} eV^{-1} (PI), 1.65 $\times 10^{13}$ cm^{-2} eV^{-1} (50 nm SiO_2) and 2.1 $\times 10^{13}$ cm^{-2} eV^{-1} (300 nm SiO_2), respectively.

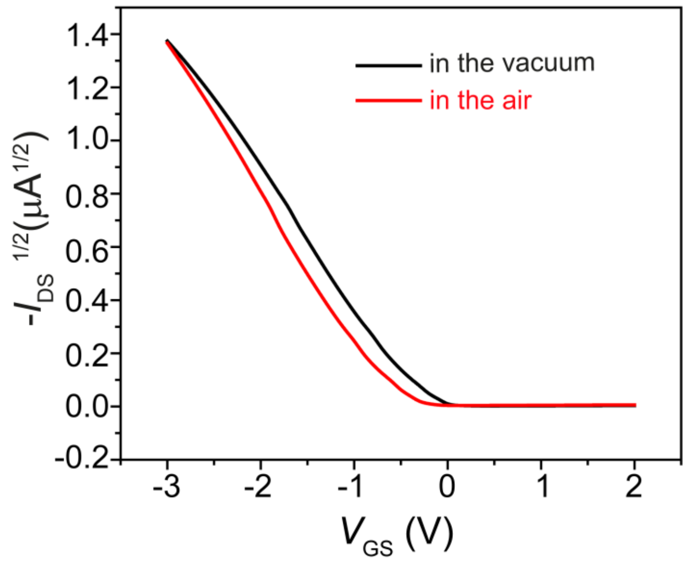

Supplementary Figure 10 | The electrical characterization of pentacene OTFTs based on copolymer dielectric in the air and in the vacuum. Typical transfer characteristics of the OTFT in the air and in the vacuum.

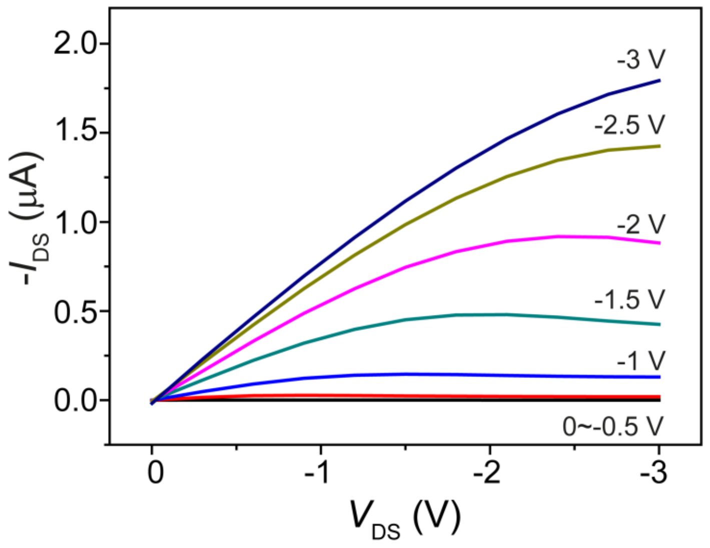

Supplementary Figure 11 | The electrical characterization of pentacene OTFTs based on copolymer dielectric. Output curves at gate voltages from 0 to -3 V in 0.5 V step.

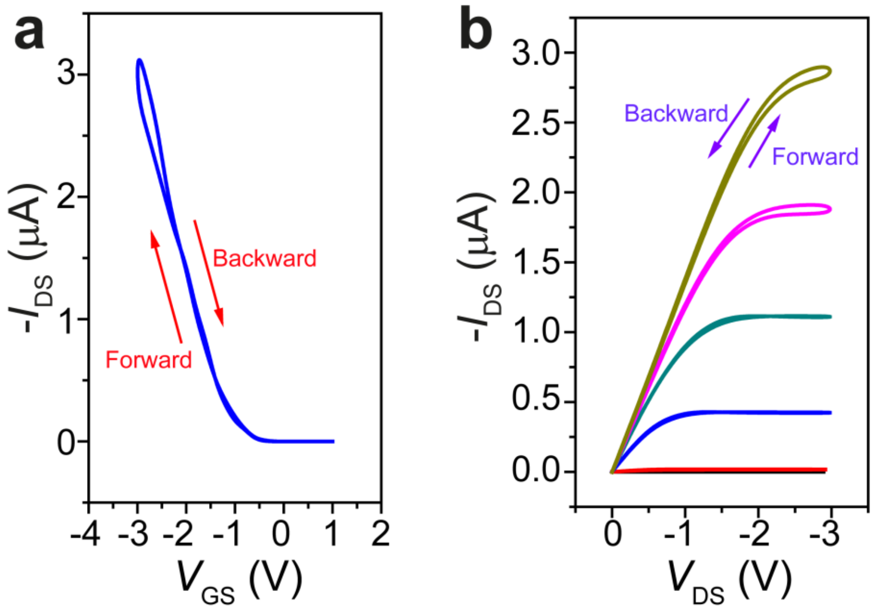

Supplementary Figure 12 | The hysteresis effect characterization of pentacene OTFTs based on copolymer dielectric. Typical (**a**) transfer curves and (**b**) output curves (both forward and reverse sweeps) of the OTFT with 50 nm pentacene and a channel dimension of W = 240 μm, L = 30 μm.

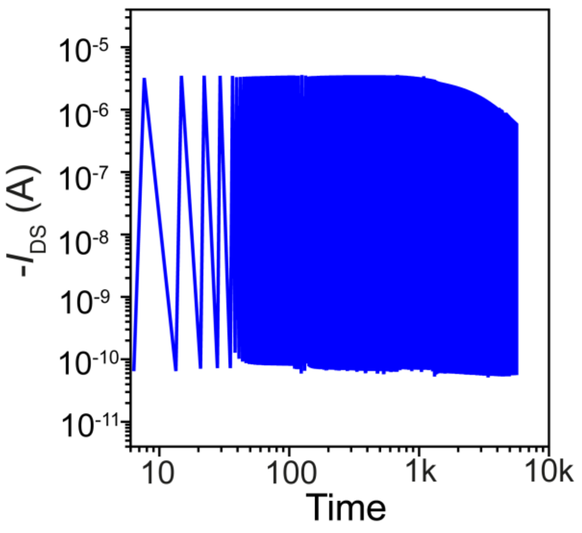

Supplementary Figure 13 | The operating stability characterization of pentacene OTFTs based on copolymer dielectric. Switching cycles of drain current as a function of cycling time.

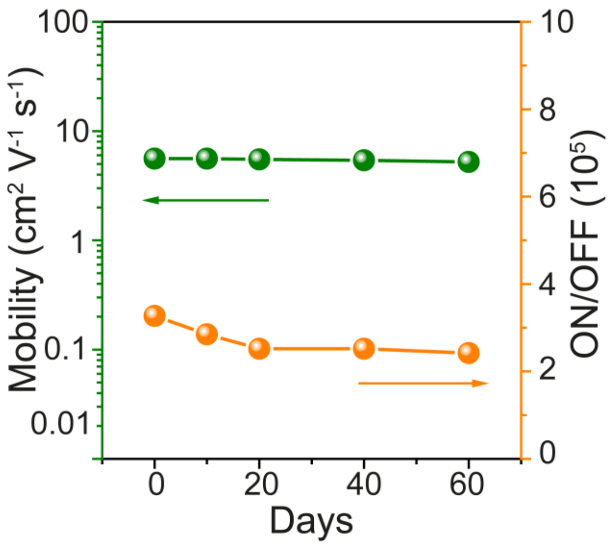

Supplementary Figure 14 | The air-stability characterization of pentacene OTFTs based on copolymer dielectric. Mobility and ON/OFF ratio as a function of time. The devices show outstanding good air stability during shelf-life tests for 60 days (not only the mobility but also the ratio of ON/OFF) and only 6% degradation of device performance was observed in all these devices.

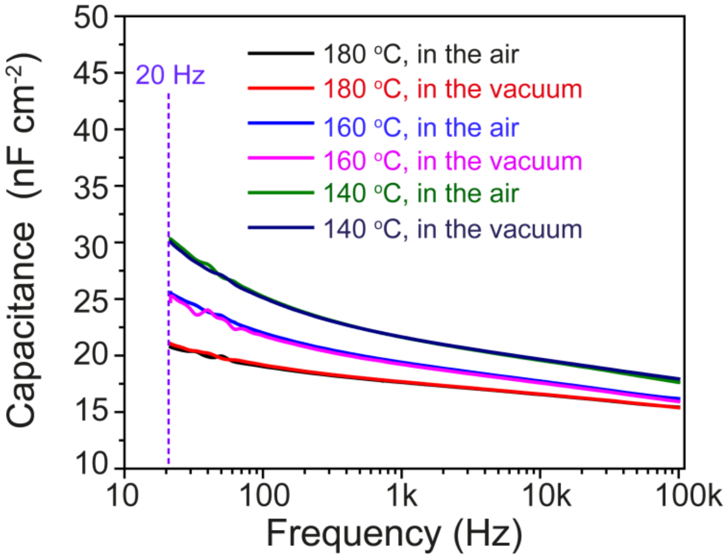

Supplementary Figure 15 | The frequency dependence of capacitance for copolymer dielectrics with different annealing temperatures. The capacitance per unit area of copolymer thin film with different annealing temperatures is measured (from 20 Hz to 100 kHz) from the sandwich structure (Au/insulator/ITO) in the air (under relative humidity of ~60%) and in the vacuum. Negligible difference of capacitance was observed in the air and in the vacuum.

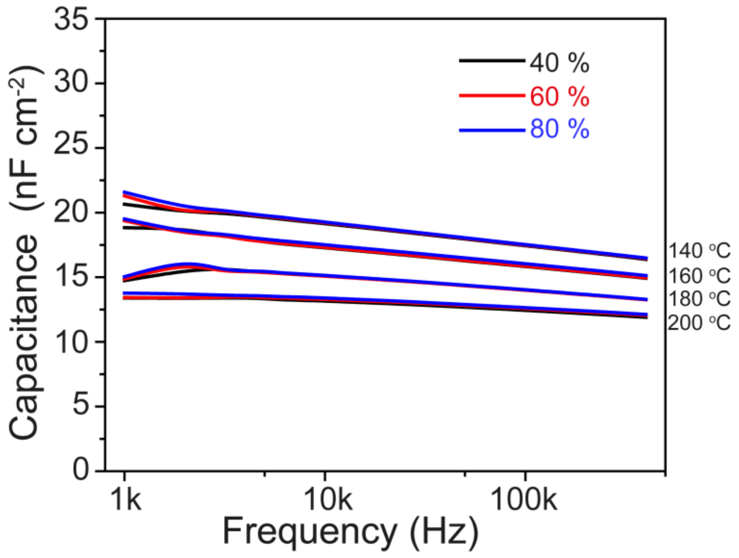

Supplementary Figure 16 | The capacitance measurement under a manual control of the relative humidity. Negligible difference of capacitance was observed under different relative humidity.

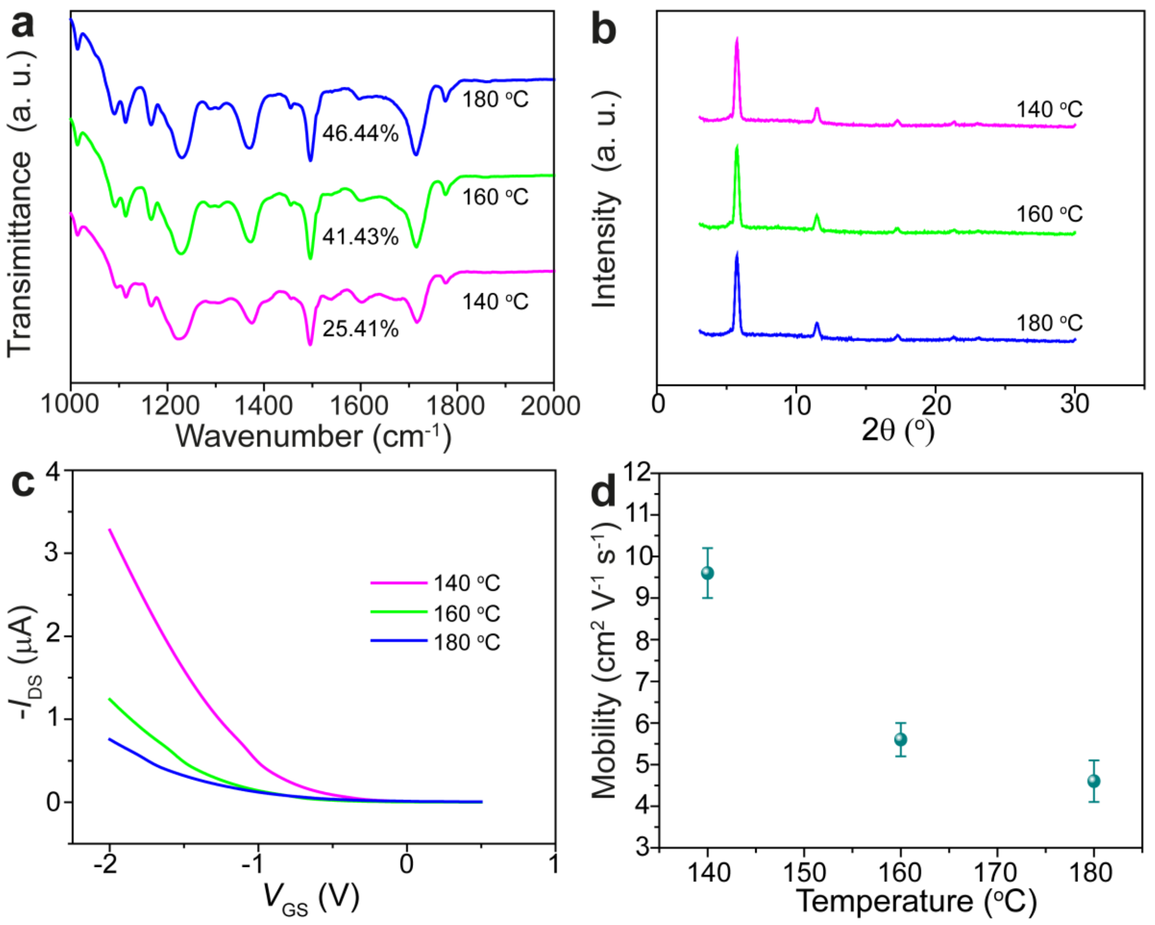

Supplementary Figure 17 | The characterization of PAA films under different annealing temperatures. (**a**) Attenuated total reflection (ATR) infrared spectroscopy of PAA with different imidization temperatures. (**b**) XRD patterns of pentacene films (50 nm) grown on these three dielectrics. (**c**) Typical transfer curves of the OTFT with 50 nm pentacene and a channel dimension of W = 240 μm, L = 30 μm. (**d**) Mobility as a function of temperature ($C_{140\,^{o}C}$, 30 nF cm^{-2}; $C_{160\,^{o}C}$, 24 nF cm^{-2}; $C_{180\,^{o}C}$, 22 nF cm^{-2}). The error bars show the standard error of the mean.

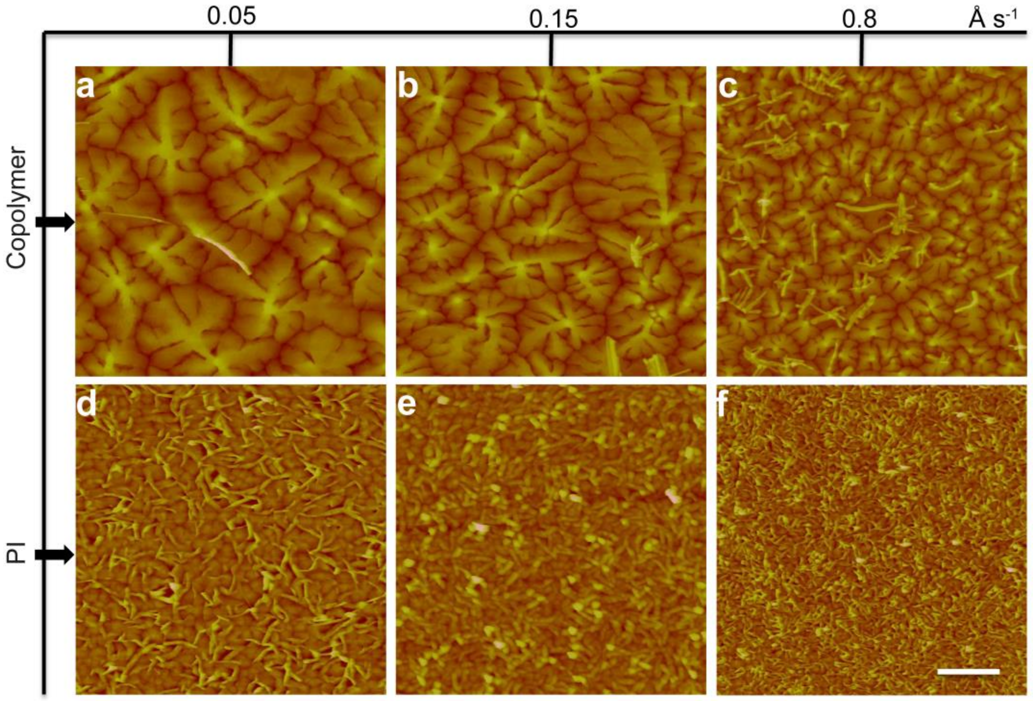

Supplementary Figure 18 | AFM characterization of pentacene films on copolymer and PI dielectric. AFM images of pentacene films deposited on the copolymer at different deposition rates: (**a**) 0.05 Å s^{-1}; (**b**) 0.15 Å s^{-1}; (**c**) 0.8 Å s^{-1}. AFM images of pentacene films deposited on the PI at different deposition rates: (**d**) 0.05 Å s^{-1}; (**e**) 0.15 Å s^{-1}; (**f**) 0.8 Å s^{-1}. Scale bar, 1 μm.

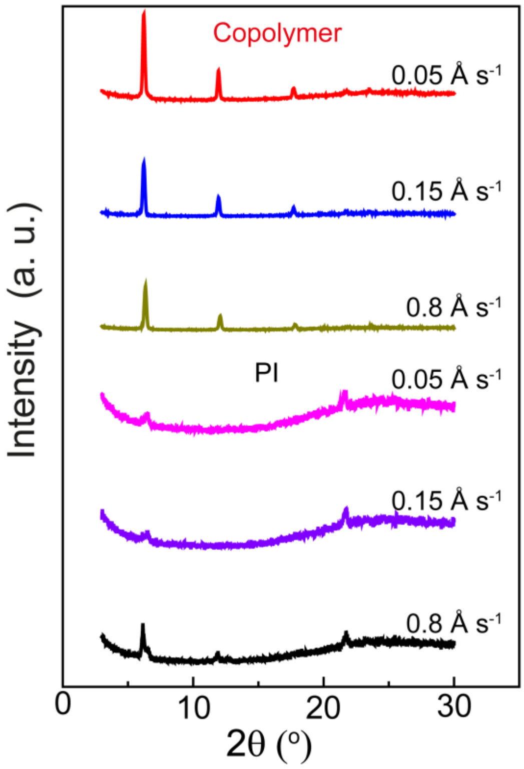

Supplementary Figure 19 | XRD patterns of pentacene films on copolymer and PI dielectric. XRD patterns of pentacene films grown on copolymer and PI with different evaporation rates.

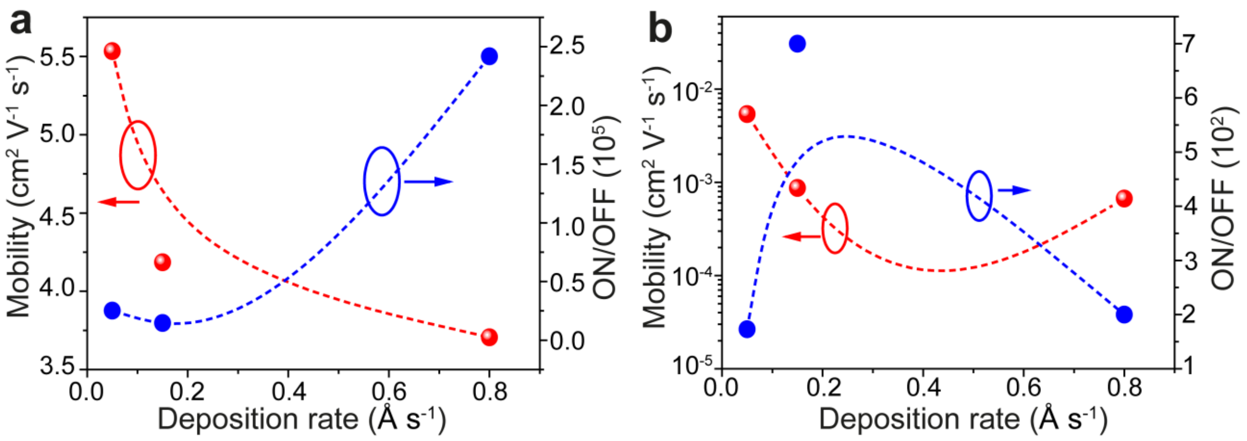

Supplementary Figure 20 | The performance of the pentacene-based OTFTs. Mobility and on/off ratio as a function of deposition rates based on (**a**) copolymer and (**b**) PI.

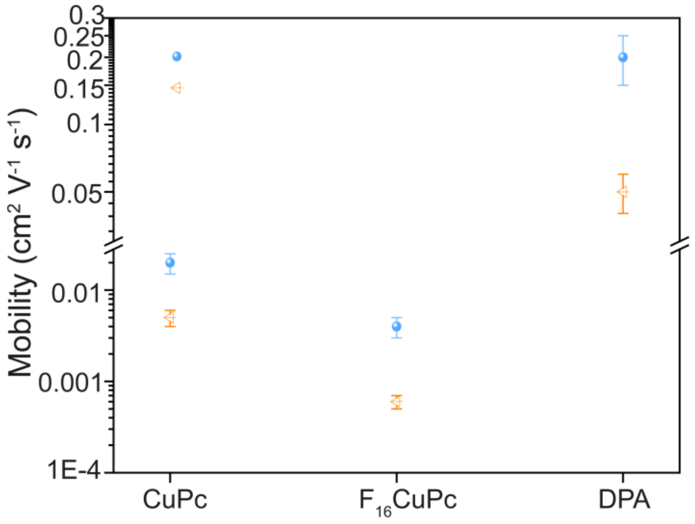

Supplementary Figure 21 | The performance of the CuPc-, F_{16}CuPc- and DPA-based OTFTs. Mobilities based on different organic semiconductors with copolymer and PI dielectrics, respectively. The error bars show the standard error of the mean.

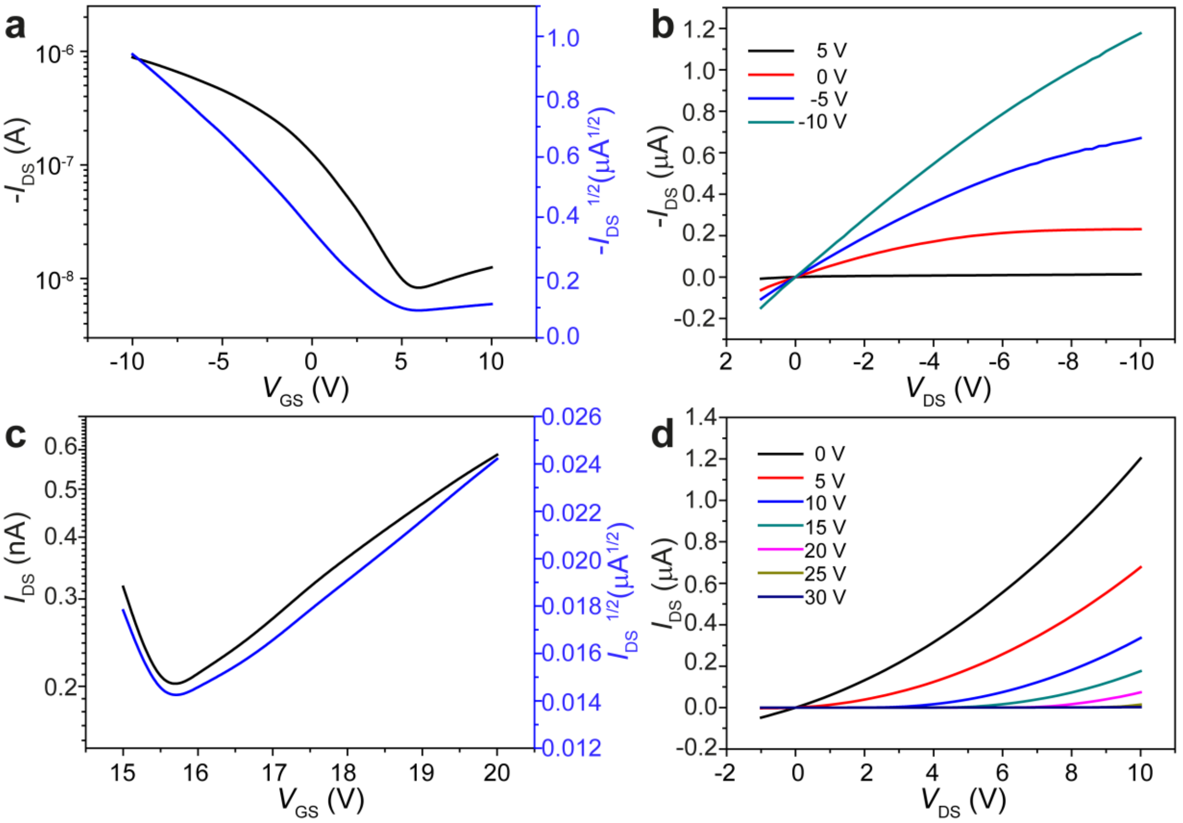

Supplementary Figure 22 | The performance of the BP3T based OTFTs. **(a, b)** Typical p-type transfers curve (V_{DS}= -10 V) of the OTFT with BP3T[1] as active layers. (**c, d**) Typical n-type transfers curve (V_{DS} = 10 V) of the OTFT with BP3T as active layers.

Supplementary Table 1. Surface energy of dielectric films and pentacene on the dielectric films.

annealing temperature (°C)	contact angle [°] Water	Ethylene glycol	γ_s [mJ m^{-2}] [b]	γ^d_s [mJ m^{-2}] [a]	γ^p_s [mJ m^{-2}] [a]
Copolymer	86.3	60.6	28.5	23.8	4.7
PI	82.7	57.4	29.4	22.9	6.5
Pentacene/ Copolymer	95.1	65.4	31.4	30.5	0.9
Pentacene/PI	103.1	63.3	50.5	49.9	0.6

[a] γ^p_s and γ^d_s are the polar and dispersion components of the surface energy, respectively. [b] γ_s is the surface energy of polyimide gate insulator. $\gamma_s = \gamma^p_s + \gamma^d_s$.

Supplementary Table 2. Summary of the reports of OTFTs with polyimide as gate dielectrics.

Semiconductor	Substrate	Thickness (nm)	$T_{annealing}$ (°C)	Mobility (cm^2 V^{-1} s^{-1})	Structure	Operating Voltage (V)	Reference
P3HT	ITO/Glass	N.A.	120	0.03	TC,BG	-30	26
Pentacene	PEN	540	180	1	TC,BG	-100	32
Pentacene	PEN	900	180	0.3	TC,BG	-40	33
Pentacene	Polyimide	500	180	0.5	TC,BG	-40	34
Pentacene	Glass	450	250	0.48	TC,BG	-50	27
Pentacene	PES	300	150	0.2	TC,BG	-50	28
Pentacene	Polyimide	1000	180	0.7	BC,BG	-100	35
Pentacene	ITO/Glass	300	200	0.36	TC,BG	-40	29
Pentacene	PEN	600	180	0.4	TC,BG	-60	36
PTCDI-C13	PEN		180	0.2	TC,BG		
Pentacene	ITO/Glass	300	160	0.16	TC,BG	-40	30
C10-BTBT	ITO/Glass	350	200	0.56	TC,BG	-60	31
Pentacene	Polyimide	800	300	0.5	BC,BG	-100	24
PDVT-10	Polyimide	1000	300	1.5	BC,BG	-80	25
Pentacene	ITO/PET	160	200	5.6	TC,BG	-3	This work

TC: Top contact; BG: bottom gate; BC: bottom contact

Supplementary Methods

The surface energy of dielectric films and pentacene on the dielectric films was evaluated by measuring the contact angles of water and ethylene glycol. The following supplementary equation[2, 3] was used to calculate the surface energy of different dielectrics. The dispersion (γ_s^d) and polar (γ_s^p) components of surface energy, and the total surface energy (γ_s) were obtained from the following supplementary equation.

$$1 + \cos\theta = \frac{2\sqrt{\gamma_s^d}\sqrt{\gamma_{lv}^d}}{\gamma_{lv}} + \frac{2\sqrt{\gamma_s^p}\sqrt{\gamma_{lv}^p}}{\gamma_{lv}}$$

The surface energy (γ_{lv}), the dispersion component (γ_{lv}^d) and the polar component (γ_{lv}^p) values used to solve this supplementary equation were, 72.2, 22.0, 50.2 mJ m^{-2} for water, and 48.0, 29.0, 19.0 mJ m^{-2} for ethylene glycol, respectively.

Supplementary References

1. Bisri, S. Z.; Takenobu, T.; Yomogida, Y.; Shimotani, H.; Yamao, T.; Hotta, S.; Iwasa, Y. High Mobility and Luminescent Efficiency in Organic Single-Crystal Light-Emitting Transistors. *Adv. Funct. Mater.* **19**, 1728-1735 (2009).
2. Michalski, M.; Hardy, J.; Saramago, B. J. V. On the Surface Free Energy of PVC/EVA Polymer Blends: Comparison of Different Calculation Methods. *J. Colloid Interface Sci.* **208**, 319-328 (1998).
3. Yang, S. Y.; Shin, K.; Park, C. E. The Effect of Gate-Dielectric Surface Energy on Pentacene Morphology and Organic Field-Effect Transistor Characteristics. *Adv. Funct. Mater.* **15**, 1806-1814 (2005).
